# Supplementary material for: Heparin-based hydrogel scaffolding alters the transcriptomic profile and increases the chemoresistance of MDA-MB-231 triple-negative breast cancer cells
Source: Biomater Sci. 2020 Feb 13;8(10):2786–96. doi: 10.1039/c9bm01481k (PMC7497406; doi:10.1039/c9bm01481k)
Supplement: Supplementary file 2 [file BM-008-C9BM01481K-s002.zip › Supplementary File 4/HP-B hydrogel + EGF (s)vControl/Pathways/my_analysis.Gsea.1545201170987/HALLMARK_PROTEIN_SECRETION.html]

Details for gene set HALLMARK\_PROTEIN\_SECRETION[GSEA]

|  || Dataset | expr.class.cls#HYDEGF\_versus\_CONTROL.class.cls#HYDEGF\_versus\_CONTROL\_repos |
| Phenotype | class.cls#HYDEGF\_versus\_CONTROL\_repos |
| Upregulated in class | CONTROL |
| GeneSet | HALLMARK\_PROTEIN\_SECRETION |
| Enrichment Score (ES) | -0.5989285 |
| Normalized Enrichment Score (NES) | -2.588551 |
| Nominal p-value | 0.0 |
| FDR q-value | 0.0 |
| FWER p-Value | 0.0 |
Table: GSEA Results Summary

  

Fig 1: Enrichment plot: HALLMARK\_PROTEIN\_SECRETION      
 Profile of the Running ES Score & Positions of GeneSet Members on the Rank Ordered List

  

| PROBE | DESCRIPTION (from dataset) | GENE SYMBOL | GENE\_TITLE | RANK IN GENE LIST | RANK METRIC SCORE | RUNNING ES | CORE ENRICHMENT || 1 | EGFR | na |  |  | 1395 | 1.470 | -0.0579 | No |
| 2 | GOSR2 | na |  |  | 1820 | 1.319 | -0.0665 | No |
| 3 | DOPEY1 | na |  |  | 1860 | 1.308 | -0.0551 | No |
| 4 | GBF1 | na |  |  | 2605 | 1.103 | -0.0827 | No |
| 5 | SGMS1 | na |  |  | 3787 | 0.849 | -0.1358 | No |
| 6 | ATP6V1B1 | na |  |  | 4023 | 0.808 | -0.1398 | No |
| 7 | ARFGEF2 | na |  |  | 4648 | 0.712 | -0.1651 | No |
| 8 | MON2 | na |  |  | 4736 | 0.700 | -0.1625 | No |
| 9 | COG2 | na |  |  | 6146 | 0.483 | -0.2313 | No |
| 10 | CLN5 | na |  |  | 6263 | 0.468 | -0.2325 | No |
| 11 | ARFGEF1 | na |  |  | 6495 | 0.437 | -0.2401 | No |
| 12 | AP2M1 | na |  |  | 7100 | 0.358 | -0.2681 | No |
| 13 | STX16 | na |  |  | 7135 | 0.354 | -0.2662 | No |
| 14 | ZW10 | na |  |  | 7329 | 0.325 | -0.2729 | No |
| 15 | YKT6 | na |  |  | 8014 | 0.241 | -0.3063 | No |
| 16 | OCRL | na |  |  | 8767 | 0.155 | -0.3440 | No |
| 17 | GOLGA4 | na |  |  | 8839 | 0.143 | -0.3463 | No |
| 18 | TSPAN8 | na |  |  | 9050 | 0.123 | -0.3560 | No |
| 19 | COPE | na |  |  | 10116 | 0.003 | -0.4117 | No |
| 20 | AP3B1 | na |  |  | 10289 | -0.020 | -0.4205 | No |
| 21 | AP2S1 | na |  |  | 10871 | -0.089 | -0.4500 | No |
| 22 | KIF1B | na |  |  | 11259 | -0.132 | -0.4689 | No |
| 23 | KRT18 | na |  |  | 11421 | -0.149 | -0.4758 | No |
| 24 | SSPN | na |  |  | 12019 | -0.219 | -0.5048 | No |
| 25 | RER1 | na |  |  | 12077 | -0.229 | -0.5054 | No |
| 26 | GNAS | na |  |  | 12371 | -0.271 | -0.5180 | No |
| 27 | NAPA | na |  |  | 12403 | -0.272 | -0.5168 | No |
| 28 | AP1G1 | na |  |  | 12613 | -0.301 | -0.5247 | No |
| 29 | VPS45 | na |  |  | 12614 | -0.301 | -0.5216 | No |
| 30 | DNM1L | na |  |  | 13067 | -0.352 | -0.5416 | No |
| 31 | PPT1 | na |  |  | 13258 | -0.379 | -0.5476 | No |
| 32 | CLTA | na |  |  | 13263 | -0.380 | -0.5440 | No |
| 33 | GLA | na |  |  | 13717 | -0.444 | -0.5631 | No |
| 34 | SCRN1 | na |  |  | 13928 | -0.475 | -0.5692 | No |
| 35 | STX7 | na |  |  | 14022 | -0.491 | -0.5690 | No |
| 36 | CAV2 | na |  |  | 14423 | -0.542 | -0.5844 | No |
| 37 | TSG101 | na |  |  | 14435 | -0.546 | -0.5793 | No |
| 38 | ATP6V1H | na |  |  | 14532 | -0.560 | -0.5786 | No |
| 39 | ARFIP1 | na |  |  | 14673 | -0.586 | -0.5799 | No |
| 40 | ERGIC3 | na |  |  | 14725 | -0.593 | -0.5765 | No |
| 41 | TOM1L1 | na |  |  | 15011 | -0.635 | -0.5849 | No |
| 42 | ARFGAP3 | na |  |  | 15121 | -0.657 | -0.5838 | No |
| 43 | SCAMP3 | na |  |  | 15272 | -0.684 | -0.5846 | No |
| 44 | AP2B1 | na |  |  | 15298 | -0.686 | -0.5789 | No |
| 45 | ANP32E | na |  |  | 15680 | -0.758 | -0.5910 | Yes |
| 46 | TMX1 | na |  |  | 15832 | -0.788 | -0.5908 | Yes |
| 47 | CTSC | na |  |  | 15844 | -0.790 | -0.5833 | Yes |
| 48 | M6PR | na |  |  | 15895 | -0.801 | -0.5776 | Yes |
| 49 | SEC31A | na |  |  | 16110 | -0.846 | -0.5801 | Yes |
| 50 | CLTC | na |  |  | 16121 | -0.850 | -0.5719 | Yes |
| 51 | VAMP4 | na |  |  | 16216 | -0.875 | -0.5678 | Yes |
| 52 | TPD52 | na |  |  | 16281 | -0.893 | -0.5620 | Yes |
| 53 | MAPK1 | na |  |  | 16445 | -0.935 | -0.5609 | Yes |
| 54 | COPB1 | na |  |  | 16525 | -0.951 | -0.5553 | Yes |
| 55 | IGF2R | na |  |  | 16595 | -0.968 | -0.5489 | Yes |
| 56 | RAB14 | na |  |  | 16677 | -0.994 | -0.5430 | Yes |
| 57 | ARF1 | na |  |  | 16848 | -1.042 | -0.5411 | Yes |
| 58 | NAPG | na |  |  | 16861 | -1.044 | -0.5310 | Yes |
| 59 | STAM | na |  |  | 16948 | -1.072 | -0.5245 | Yes |
| 60 | RAB5A | na |  |  | 17114 | -1.116 | -0.5217 | Yes |
| 61 | SNX2 | na |  |  | 17231 | -1.154 | -0.5159 | Yes |
| 62 | VAMP7 | na |  |  | 17289 | -1.178 | -0.5067 | Yes |
| 63 | SEC22B | na |  |  | 17528 | -1.269 | -0.5062 | Yes |
| 64 | SEC24D | na |  |  | 17563 | -1.283 | -0.4947 | Yes |
| 65 | TMED2 | na |  |  | 17755 | -1.362 | -0.4907 | Yes |
| 66 | RPS6KA3 | na |  |  | 17760 | -1.363 | -0.4769 | Yes |
| 67 | GALC | na |  |  | 17768 | -1.368 | -0.4632 | Yes |
| 68 | ABCA1 | na |  |  | 17887 | -1.432 | -0.4547 | Yes |
| 69 | CLCN3 | na |  |  | 17982 | -1.478 | -0.4444 | Yes |
| 70 | ARCN1 | na |  |  | 17997 | -1.483 | -0.4299 | Yes |
| 71 | USO1 | na |  |  | 18109 | -1.553 | -0.4197 | Yes |
| 72 | ADAM10 | na |  |  | 18162 | -1.589 | -0.4061 | Yes |
| 73 | SCAMP1 | na |  |  | 18204 | -1.603 | -0.3918 | Yes |
| 74 | CD63 | na |  |  | 18241 | -1.632 | -0.3768 | Yes |
| 75 | COPB2 | na |  |  | 18316 | -1.697 | -0.3633 | Yes |
| 76 | PAM | na |  |  | 18328 | -1.699 | -0.3464 | Yes |
| 77 | ICA1 | na |  |  | 18335 | -1.703 | -0.3292 | Yes |
| 78 | AP3S1 | na |  |  | 18338 | -1.703 | -0.3118 | Yes |
| 79 | RAB22A | na |  |  | 18393 | -1.749 | -0.2966 | Yes |
| 80 | YIPF6 | na |  |  | 18415 | -1.767 | -0.2795 | Yes |
| 81 | SNAP23 | na |  |  | 18457 | -1.805 | -0.2631 | Yes |
| 82 | ATP1A1 | na |  |  | 18531 | -1.860 | -0.2478 | Yes |
| 83 | RAB9A | na |  |  | 18608 | -1.935 | -0.2319 | Yes |
| 84 | VPS4B | na |  |  | 18613 | -1.943 | -0.2121 | Yes |
| 85 | RAB2A | na |  |  | 18694 | -2.039 | -0.1953 | Yes |
| 86 | VAMP3 | na |  |  | 18776 | -2.153 | -0.1774 | Yes |
| 87 | LAMP2 | na |  |  | 18797 | -2.190 | -0.1559 | Yes |
| 88 | LMAN1 | na |  |  | 18876 | -2.320 | -0.1362 | Yes |
| 89 | SOD1 | na |  |  | 18910 | -2.370 | -0.1135 | Yes |
| 90 | TMED10 | na |  |  | 18916 | -2.384 | -0.0893 | Yes |
| 91 | ATP7A | na |  |  | 18940 | -2.432 | -0.0655 | Yes |
| 92 | BET1 | na |  |  | 18946 | -2.453 | -0.0405 | Yes |
| 93 | BNIP3 | na |  |  | 18959 | -2.482 | -0.0156 | Yes |
| 94 | STX12 | na |  |  | 19045 | -2.728 | 0.0080 | Yes |
Table: GSEA details [plain text format]

  

Fig 2: HALLMARK\_PROTEIN\_SECRETION      
 Blue-Pink O' Gram in the Space of the Analyzed GeneSet

  

Fig 3: HALLMARK\_PROTEIN\_SECRETION: Random ES distribution      
 Gene set null distribution of ES for **HALLMARK\_PROTEIN\_SECRETION**
